# Systemic AA Amyloidosis Secondary to Chronic Bronchiectasis and Primary Immunodeficiency: A Case Report

**DOI:** 10.7759/cureus.88790

**Published:** 2025-07-26

**Authors:** Juhi Jain, Muhammad Junaid Akram, Gerald Langman, Rahul Mukherjee

**Affiliations:** 1 Respiratory Medicine, University Hospitals Birmingham NHS Trust, Birmingham, GBR; 2 Histopathology, University Hospitals Birmingham NHS Trust, Birmingham, GBR

**Keywords:** bronchiectasis, end-stage renal disease, pseudomonas aeruginosa, secondary amyloidosis, serum amyloid a

## Abstract

A 37-year-old ex-teacher, having suffered severe bronchiectasis for over a decade and not diagnosed as having primary specific antibody deficiency, later developed systemic AA amyloidosis and end-stage renal disease. Persistent respiratory symptoms developed at age 26, and subsequent investigations revealed cylindrical bronchiectasis and impaired specific antibody responses to *Streptococcus pneumoniae* and *Haemophilus influenzae*. Lack of compliance with the treatment and follow-up despite continuous specialist treatment led to a gradual decline in lung function and frequent *Pseudomonas aeruginosa* and *Streptococcus pneumoniae*-induced infections. The patient has progressively developed extreme systemic complications with severe renal dysfunction and hypoalbuminemia. In May 2024, a biopsy of the kidney indicated that the amyloid A was deposited all over the kidneys, and the condition was identified as amyloidosis caused by an underlying chronic respiratory disease. By May 2025, his renal functions had deteriorated significantly, leading to renal replacement therapy, as well as extreme malnutrition and frequent infections. The given case demonstrates the importance of prompt and thorough treatment of chronic inflammatory diseases and their etiologic alterations in immune disorders to prevent severe complications, such as AA amyloidosis.

## Introduction

Amyloidosis is a complex group of diseases characterized by the extracellular deposition of insoluble protein fibrils in various tissues and organs, ultimately leading to progressive organ failure [1]. AA amyloidosis is a secondary type that typically occurs as a sequel to chronic inflammatory or infectious illnesses, such as rheumatoid arthritis, inflammatory bowel disease, or chronic infections like tuberculosis and bronchiectasis [2]. Under such circumstances, dysregulated production of serum amyloid A (SAA), which is produced during the acute-phase reaction, persists and accumulates in the form of amyloid fibrils as a result of improper folding. Moreover, in patients with bronchiectasis, primary immunodeficiencies increase their risk of frequent and recurrent infections, which are characteristic of the condition, including those that target antibody production [3]. This risk factor exacerbates the inflammatory process, increasing the likelihood of amyloidosis. A decreased antibody response to polysaccharide antigens accompanies normal levels of immunoglobulins in specific antibody deficiency, leading to recurrent respiratory infections [4].

This case report demonstrates how the interactivity of chronic inflammation and an unknown immune deficiency resulted in systemic AA amyloidosis in the form of end-stage renal disease (ESRD) in a young male with a long-term history of poorly managed bronchiectasis. This case indicates that timely diagnosis, treatment, and close observation of the course of the chronic inflammatory disease are paramount, and immune system disorder is the most critical priority that should be avoided by such development of events; thus, an early diagnosis, the need to monitor renal functioning, and proteinuria are of high consideration.

## Case presentation

A 37-year-old former teacher of Asian origin with a longstanding history of low body weight since 2008 presented for the first time to respiratory services in March 2014. His chief complaints were the presence of a productive cough, which was persistent, and intermittent fever and nocturnal sweats. He experienced only partial relief with the antibiotics. He was unemployed when he first presented with symptoms. His prior medical history was unremarkable, and he had no social history of smoking or drinking. He had no known drug allergies and was not taking any routine medications. His background did not reveal anything much except that his brothers had mild asthma. He had no history of exposure to tuberculosis, work-related exposure to pollutants, or pet allergies. Characteristically, he had a slim build and was of low weight; this persisted throughout his clinical progression. Regardless of nutritional support and boosted with high-calorie diets, he weighed 45 kg (BMI 15 kg/m^2^) in November 2014 and had a further decline in weight, reaching 41.6 kg (BMI 14.4 kg/m^2^) in May 2017.

**Figure 1:**
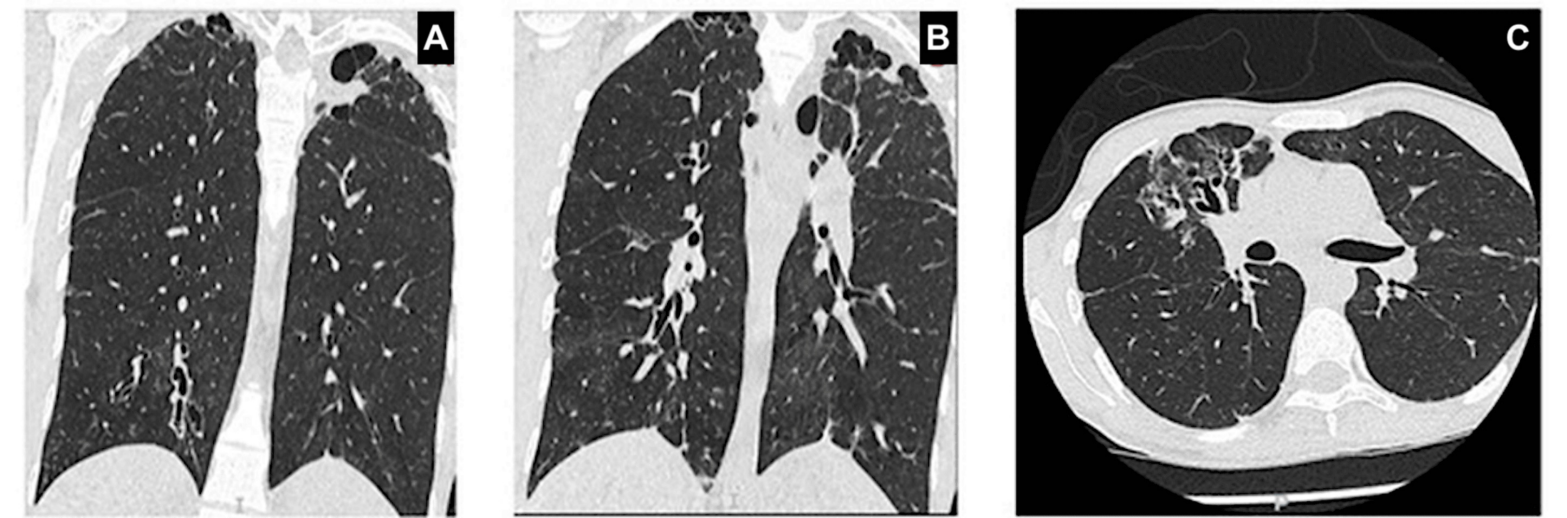
CT of the thorax demonstrating initial findings of structural lung disease (images represent the baseline CT findings, consistent with early bronchiectatic changes and inflammatory activity) (A) Coronal CT image showing left apical bullae formation; (B) coronal CT showing left apical bullae with evidence of cylindrical bronchiectasis, more pronounced in the right upper and middle lobes; (C) axial CT section demonstrating right-sided cylindrical bronchiectasis associated with ground-glass opacities, suggestive of active inflammation or infection.

By the middle of 2025, his BMI had dropped below 15 kg/m^2^. While his vital signs, including pulse, blood pressure, temperature, and respiratory rate, were recorded within normal limits on his outpatient appointments, the only consistent finding on physical examination was digital clubbing. The chest examination on several occasions showed impaired air entry accompanied by mild crackles on both sides. Bilateral shadowing in the upper zone of the chest was observed on the first chest radiography but remained unchanged by subsequent secondary radiography. A CT of the thorax was subsequently performed, which showed bilateral cylindrical bronchiectasis, mainly in the upper lobes, patchy ground-glass changes, and apical emphysematous bullae (Figure 1).

**Figure 2:**
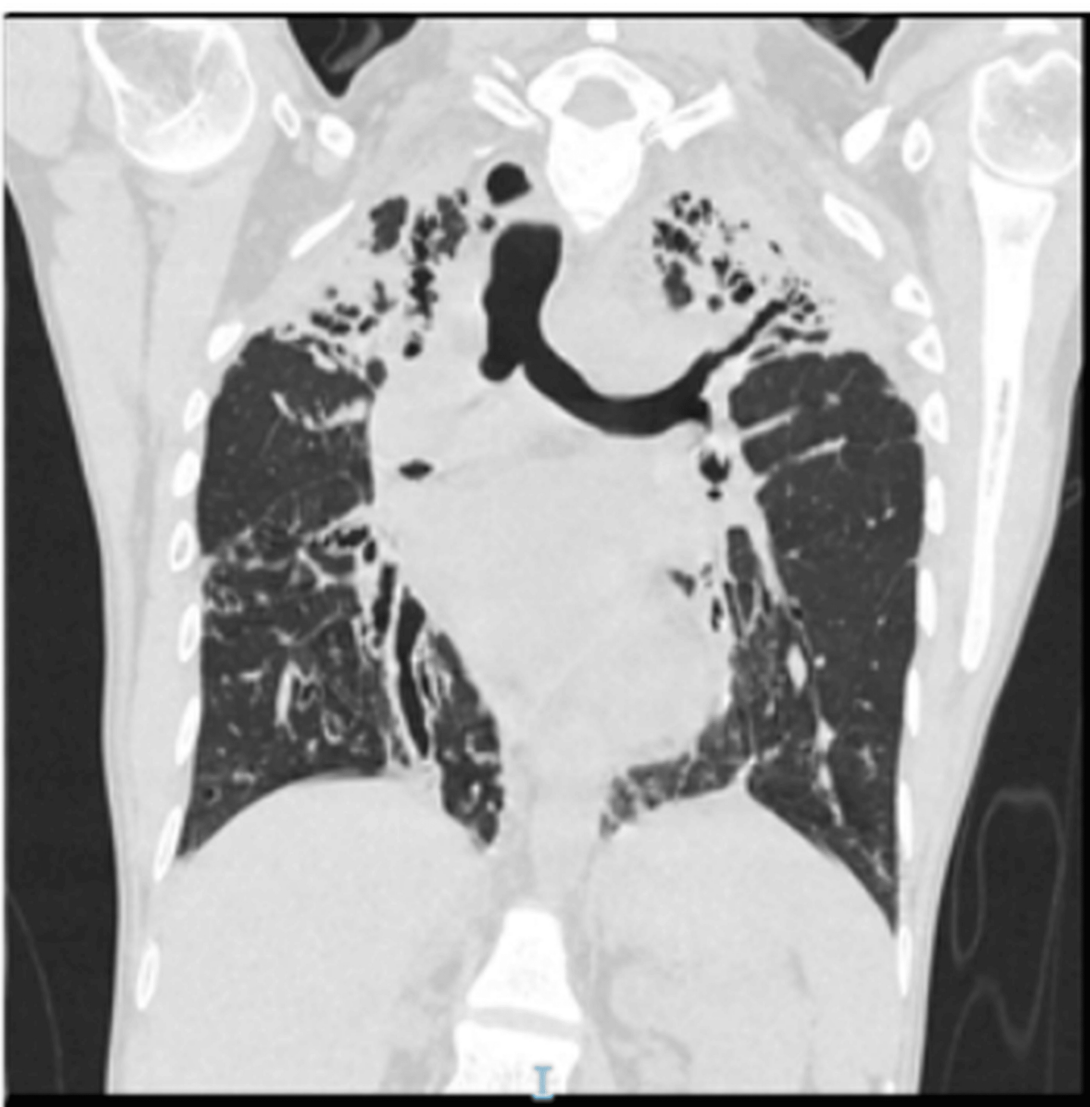
High-resolution CT of the chest revealing interval progression The follow-up high-resolution CT of the chest shows marked progression of bilateral cylindrical bronchiectasis with worsening mucous plugging and increased distribution of infiltrates. Varicose bronchiectasis was noted in the right lung, particularly in the lower lobes, indicating architectural distortion and chronicity. Compared to Figure 1, this scan shows an interval worsening with an increased extent of bronchiectasis and more prominent parenchymal changes, including denser infiltrates and volume loss, most notably on the right side.

A subsequent chest CT confirmed the radiological progression of bilateral scarred upper lobes and right middle lobe varicose bronchiectasis (Figure 2). With a given clinical course and a bronchiectasis severity index score of 13, extensive blood examinations were conducted to determine the underlying etiology, which included immunoglobulins and specific antibody levels for *Haemophilus influenzae* type B, tetanus, and pneumococcus. Markedly low titers to particular antibodies against pneumococcus and *Haemophilus influenzae* were identified, and it was highly likely that there was a defective immunological response. Serology of HIV, hepatitis C, and T-Spot IGRA (interferon-gamma release assay) of tuberculosis all resulted negative. His bronchoscopy with bronchoalveolar lavage was also reported to be negative for tuberculosis and other bacterial and fungal infections. Cystic fibrosis was systematically excluded by normal sweat test results (24 mmol/L) and negative CF genetics, and primary ciliary dyskinesia was also ruled out via screening. By April 2017, the diagnosis was definitively established as bronchiectasis affecting both upper lobes and the right middle lobe of unknown etiology, coupled with probable specific antibody deficiency.

Throughout his clinical course, the patient experienced a progressive and notable decline in lung function, with spirometry consistently demonstrating severe obstruction. Initially, his FEV1 was 1.53 L (37% predicted) in November 2014, which progressively declined to 1.28 L (31% predicted) by June 2015, 0.98 L (31% predicted) by December 2017, and ultimately reached 1.00 L (24% predicted) by December 2022. Overall, his oxygen saturation remained between 95% and 98% on room air.

In view of recurrent chest infections, we had sputum microbiology on several occasions. His sputum cultures isolated various pathogens, including *Haemophilus parainfluenzae* in July 2015, *Pseudomonas aeruginosa* in May 2016 and December 2021, and *Streptococcus pneumoniae* in December 2017. His microbiology results guided antibiotic therapy during exacerbations. New symptoms occurring after 2022 included hemoptysis and hearing impairment, both of which were managed conservatively.

**Figure 3:**
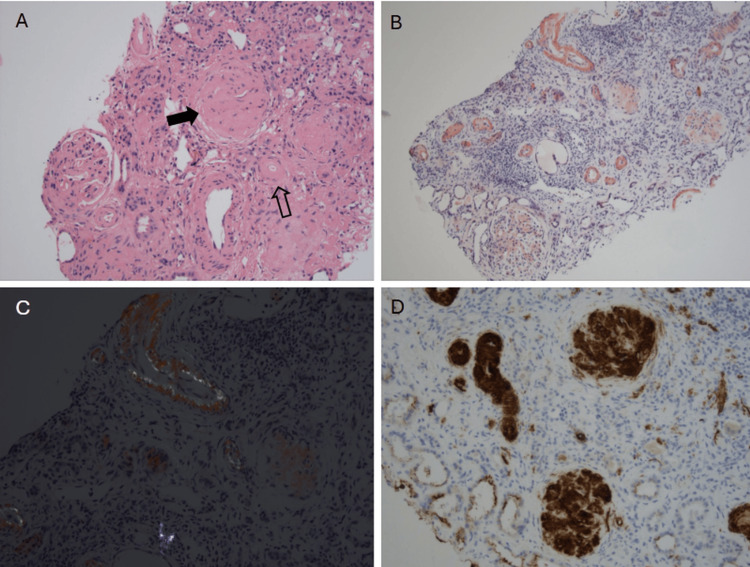
Renal biopsy (A) Homogenous eosinophilic material is present in the glomerulus (black arrow) and small artery (open arrow) (H&E, ×200). (B) This shows a brick red color on the Congo red stain (CR, ×100). (C) An apple-green birefringence is observed when examined under polarized light (CR, ×200). (D) Immunohistochemistry for anti-amyloid A antibody shows positive staining in the glomeruli and vessels (anti-AA, ×200).​ H&E: hematoxylin and eosin; CR: Congo red.

After 2022, the patient was lost to follow-up until 2024, when he presented with severe renal impairment. The patient was urgently referred to nephrology, who suggested a renal biopsy, which subsequently revealed amyloid A deposits in the kidney, confirming the underlying diagnosis of amyloidosis (Figure 3), leading to ESRD requiring regular hemodialysis. His physical status progressively worsened, with a serum albumin level of 19 g/L, reflecting severe malnutrition and persistent systemic inflammation.

**Figure 4:**
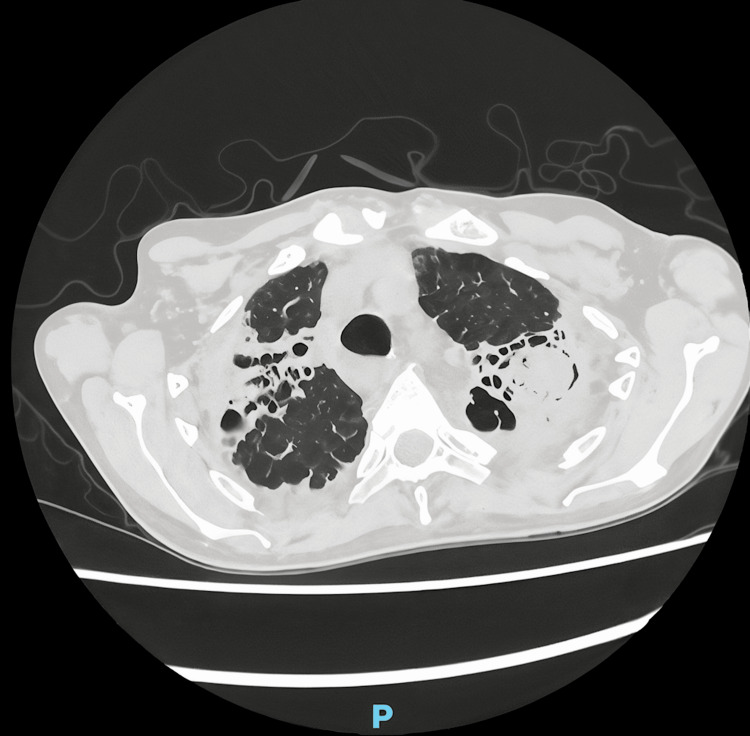
High-resolution CT scan The image shows a left-sided aspergilloma and right-sided worsening varicose bronchiectasis.

The patient presented in the bronchiectasis clinic in April 2025, and further investigations are required due to a significant fungal load within the bronchiectatic lung, with Aspergillus IgE levels exceeding 200 mg/L (normal range <2 mg/L), consistent with a probable aspergilloma (Figure 4). He was also examined to support the diagnosis of systemic amyloidosis; an electrocardiogram (ECG) showed a corrected QT interval of 445 ms, left axis deviation, and new T-wave inversions in leads III and aVF. B-type natriuretic peptide (BNP) was remarkably high at 8000 pg/mL (normal <100 pg/mL), and the serum creatine kinase was mildly raised. Echocardiography, however, did not reveal the presence of amyloid in the heart and had a retained left ventricular ejection fraction of 60%. The personality changes started in December 2024, and the brain MRI reported no cerebral signs of amyloid.

Appropriate management strategies, such as pneumococcal and *Haemophilus influenzae* type B/meningococcal C vaccinations, carbocisteine for productive cough, prophylactic oral antibiotics to prevent exacerbations, high-calorie nutritional supplements to build muscle and improve BMI, and physiotherapy for chest clearance, are commenced. A trial of itraconazole has been planned to address the underlying probable aspergilloma and fungal burden. However, patient compliance was noted to be challenging throughout his long clinical course. We have the patient booked for further follow-up with the intention of referring him to a specialized amyloid center and to ensure that his underlying, complicated bronchiectasis is appropriately managed.

## Discussion

The trajectory of this patient's disease took an astonishingly severe and atypical turn. The underlying undiagnosed primary specific antibody deficiency has significantly augmented the pathology, which ultimately triggered the aggressive systemic amyloidosis and its catastrophic multi-organ involvement, which regrettably culminated in ESRD. This particular case strongly suggests that an overlooked primary immunodeficiency can play a substantial, even accelerating, role in driving chronic inflammation and subsequent amyloid deposition. It is well established that chronic inflammation, a hallmark of bronchiectasis, serves as a significant risk factor for AA amyloidosis [3].

The patient's initial presentation with recurrent chest infections, followed by the diagnosis of extensive bronchiectasis and an impaired specific antibody response to *Streptococcus pneumoniae* and *Haemophilus influenzae*, painted a clearer picture of a specific immune deficiency. Such deficiencies are well known to impair immune responses against common encapsulated bacteria, which leads to persistent tissue damage and airway inflammation [4]. Year after year, these recurrent bacterial infections (with *Pseudomonas aeruginosa* being a notable culprit) continually stoked the inflammatory fires, resulting in persistently elevated SAA levels. Inevitably, this constant production led to the misfolding and accumulation of these proteins into amyloid fibrils.

The diagnostic route in this case effectively highlights the stealthy nature of AA amyloidosis and underscores the urgent need for diligent observation in patients with chronic inflammatory conditions, particularly since AA amyloidosis often evades early detection [5]. The classic kidney issues linked to AA-related amyloidosis, protein loss, and impaired function were all too evident in this patient, tragically leading to the life-threatening necessity of long-term renal replacement therapy for ESRD. Interestingly, despite no abnormalities on the MRI of the brain, some unusual developments occurred in December 2024: the manifestation of neuropsychiatric symptoms. Although most cases of AA amyloidosis affect systemic organs, the known neurological manifestations are highly unusual for AA amyloidosis; amyloid neuropathies are much more commonly observed with amyloid transthyretin (ATTR) and, in rare cases, with AL amyloidosis. This distinct and independent clinical presentation can be followed by further investigation, including perhaps more specific neurological exams or perhaps a reconsideration of other amyloid types in case symptoms change or become even more severe.

The heart conditions also provide valuable insights. A corrected QT of 445 ms, left axis deviation, and new T-wave inversions on ECG, along with a preserved ejection fraction on echo and elevated B-type natriuretic peptide (BNP), certainly sound like an alarm gun for possible cardiac amyloidosis. Nevertheless, given that the echocardiography did not show any amyloid deposits, the diagnosis remains unclear and necessitates further investigations. Since systemic amyloidosis may ultimately affect the heart, additional diagnostic tools such as cardiac magnetic resonance (CMR) imaging or technetium-99m pyrophosphate (PYP) scintigraphy would be essential to confirm this [6]. These would conclusively exclude the possibility of cardiac involvement, as amyloidosis in the cardiac muscles may not initially affect cardiac function and can be easily overlooked in its early stages.

Certainly, it could be one of the most important take-home messages of this case: the implication of not adhering to treatment by the patient. Such non-compliance certainly sparks the uncontrolled course of his chronic infections and the severe chronic systemic disease. It strongly highlights the importance of a thorough education for both the patient and their family. Additionally, a truly collaborative, multidisciplinary team approach, along with more robust follow-up strategies, is needed to reinforce adherence in individuals struggling with complex and chronic conditions [7].

In summary, this case vividly illustrates the aggressive and relentless course of a respiratory disease leading to AA amyloidosis, directly resulting from a poorly managed long-standing bronchiectasis combined with an underlying primary immunodeficiency. It also dramatically emphasizes the paramount importance of early detection and prompt management of primary immunodeficiencies and chronic inflammatory conditions to prevent the catastrophic consequences of systemic amyloid deposition [8]. Moreover, this case also brings to attention the inherent diagnostic complexities of amyloidosis and highlights the crucial role of a multidisciplinary approach in managing such challenging cases. An early and effective diagnosis, along with ongoing care, truly requires collaboration across various medical specialties to avoid irreversible damage.

## Conclusions

Treatment of bronchiectasis has been modernized, and this has finally proved useful in controlling inflammation and infection, thereby reducing systemic complications such as secondary amyloidosis. Nonetheless, due to patient non-compliance with treatment, this is unfortunately lost, as illustrated by this case. The undiagnosed primary specific antibody deficiency and repeated infections with different organisms in this patient caused his prolonged and poorly controlled chronic inflammation to become exaggerated.

The two different bacteria (*Pseudomonas aeruginosa* and *Streptococcus pneumoniae*) eventually promoted a persistent inflammatory process leading to amyloid deposition. This can be directly attributed to the disastrous result in ESRD as identified by renal biopsy in the amyloid A deposition that obtained a widespread distribution. Patients considered to be at higher risk of bronchiectasis should be actively screened and treated vigorously for their infections. Moreover, it is important to promote patient compliance and regularly monitor secondary amyloidosis to prevent such a disaster. This case significantly demonstrates the transformative role played by intractable inflammation and immune deficiency, making it extremely crucial to achieve timely and successful diagnosis, as well as further multidisciplinary management of the condition, to avoid irreparable organ damage. Additionally, patient compliance with treatment and regular follow-up will remain a crucial factor in the early identification and timely management of this catastrophic complication.
